# The Effect of Alterations in Consciousness on Quality of Life (QoL) in Epilepsy: Searching for Evidence

**DOI:** 10.3233/BEN-2011-0321

**Published:** 2011-03-29

**Authors:** Andreas Charidimou, Caroline Selai

**Affiliations:** UCL–Institute of NeurologyQueen SquareLondonUK

**Keywords:** Quality of life, consciousness, seizures, epilepsy, AEDs

## Abstract

The impact of epilepsy on Quality of Life (QoL) is well-documented. The ability of epileptic seizures to alter the conscious states of patients is also well established. Although there is much research on the QoL of people with epilepsy, few researchers have looked specifically at the effect of sudden, unanticipated alterations of consciousness on QoL. This lack of systematic studies of consciousness alterations and QoL in epilepsy limits our ability to shed light on this interrelation. In this article, with these limitations in mind, we focus on studies of newer AEDs. We review the evidence as to whether a significant reduction (typically more than 50%) in seizures that induce alterations of consciousness, as a result of switching to one of the newer AEDs, leads to improvements in QoL. We draw on this literature to speculate on the relationship between ictal consciousness alterations and poor QoL in epilepsy, to identify contributory and confounding factors and to highlight implications for future research. We suggest that an understanding of how factors associated with consciousness impairment affect QoL could help the treatment and management of these patients.

## 1. Introduction

With an incidence of around 50–80 cases per 100,000 persons per year, epilepsy is the commonest neurological condition, affecting people of all ages, races and social groups [17,23,43]. Epilepsy can profoundly affect many aspects of Quality of Life (QoL) [4]. Patients with epilepsy have poorer QoL than people without epilepsy or people with chronic illnesses other than epilepsy [52,60,62,65]. A key feature of epilepsy is its ability to alter the conscious states of the patients during seizures. The spectrum of alterations of consciousness can be characterised by both the patient's level of awareness and the subjective contents of consciousness [12]. The assessment of consciousness is of key significance to the phenomenological description, classification and diagnosis of epileptic seizures [20] and is reflected in the classification of epileptic seizures by the International League Against Epilepsy [8,50]. In this scheme seizures are divided into generalized and partial. The former arise from large cortical areas in both hemispheres simultaneously and consciousness is always lost, while the latter emanate from specific cortical loci in one hemisphere, and alterations of consciousness differentiate them as either simple or complex partial seizures.

Consciousness is operationally difficult to define [69]. Although there is much research on the QoL of people with epilepsy, few researchers have looked specifically at the effect on QoL of sudden, unanticipated alterations of consciousness. In this paper we review the relationship between epilepsy, impairment of consciousness and QoL. First, we define consciousness and summarise some key findings in the field of QoL in epilepsy. We then present the findings of a systematic review of published studies of newer anti-epileptic drugs (AEDs), focussing on the relationship between significant seizure reduction (and thus reduced episodes of altered conscious states) and QoL.

ISSN 0953-4180/11/$27.50 © 2011 – IOS Press and the authors. All rights reserved

### 1.1. Epilepsy and altered conscious states: A multifaceted spectrum

The dramatic loss of consciousness during a generalized tonic-clonic or other generalized seizure, is the most striking example of an alteration of conscious state and when neurologists refer to 'loss' or 'impairment' of consciousness, it is this picture that dominates [20]. However, this extreme example does not capture the complexity of consciousness and the full range of possible degrees (level and content) of impairment of consciousness in the context of an epileptic seizure (also referred as the 'ictal state') [12].

The level-content distinction of consciouness is useful when considering seizure-induced alterations of the conscious state [11,12]. Both the level and the subjective contents (the subjective experience at any given time e.g. emotions, feelings, sensations, memories and intentions) of consciousness can change over time to different degrees, according to the type of seizures being experienced [12,48]. The hallmark of generalized seizures is the loss (disappearance) of both the level and the content of consciousness; the patient is unresponsive and lacks any ictal subjective experiences [11]. On the other hand, complex partial seizures can lead to varying degrees of alterations in both level of consciousness and in the content of the ictal experience [32]. Most complex partial seizures originating in the temporal lobe are characteristically associated with specific changes in the subjective aspect of the ictal experience [21,48]. These alterations in the content of ictal consciousness may comprise mnemonic (e.g. inappropriate recall of a past event and/or a feeling of familiarity), affective (e.g. fear or happiness) or even complex perceptual phenomena (for example complex visual or auditory illusions and hallucinations, e.g. perception of visual scenes or hearing music) [21, 36]. Even simple partial seizures, which are thought to leave consciousness intact, may at times affect aspects of the contents of consciousness e.g. mnemonic, visual, auditory or somatosensory experiences [12,36]. Epilepsy-induced impairment of consciousness can go beyond the ictal conscious states to include peri-ictal phenomena, such as the confusion following a generalized tonic-clonic convulsive episode that can last from minutes up to hours and the 'epileptic aura' preceding a complex partial seizure of temporal lobe origin [1].

### 1.2. Epilepsy and QoL: Some perspectives

In recent years, there has been a surge of research into the impact of epilepsy and its treatment on QoL [24]. Earliest population health indices, used readily available numerical indicators such as mortality rates [46]. Rising expectations in recent years have led to a shift away from viewing health in terms of survival to a phase of defining it in terms of freedom from disease, to ability to perform daily activities and the current emphasis on positive themes of happiness, social and emotional well-being, and quality of life [46]. The subjective evaluation of Quality of Life (QoL) or 'health-related QoL' (HRQoL), is now a well established component of the assessment of patients suffering from chronic diseases. The field of QoL is flourishing with many robust and validated instruments, both generic and disease-specific.

QoL is regarded as an important outcome measure in epilepsy research and care, and many epilepsy-specific instruments have been developed, such as Quality of Life in Epilepsy (QOLIE-89) [42]. QoL is a multidimensional construct and most measures have a number of subscales assessing physical, psychological and social well-being. Research has consistently demonstrated that many aspects of well-being and everyday life are compromised in epilepsy. People with epilepsy experience a range of health-related QoL problems from psychological sequelae to stigma and social problems, impact on employment status and career opportunities, family life and cognitive function.

In the last two decades, QoL has become an important component in the comprehensive assessment of outcome in clinical trials of epilepsy treatments [5]. Several recent studies of the newer AEDs have included QoL as a secondary outcome measure, and the use of validated QoL instruments has yielded a wealth of robust data.

### 1.3. Assembling the pieces of the puzzle: Epilepsy, Consciousness alterations and QoL

Consciousness, perhaps the most mysterious achievement of the brain, is hard to operationally define. Consciousness: the awareness of a 'self' that is knowing, perceiving, and capable of organizing our actions and memories, is pivotal to the human flavour of everyday experience. The spectrum of seizure-induced consciousness alterations, and the impact on well-being, is of interest to the QoL researcher.

Many aspects of epilepsy which have an impact on QoL are directly related to impairment of consciousness. Epilepsy-related accidents and injuries, such as falls, burns and driving accidents, are frequently caused by consciousness alterations during seizures [22,41,61,

67]. Altered conscious states during seizures may influence almost all psycho-social problems ranging from work and family disability, to social stigma [30], lost time and cognitive disturbances. Vickrey et al have shown that the amount of time that adult patients spend before returning to normal after seizures, has a strong correlation with poor QoL [63]. This time before the patient is completely "back to normal", represents a period of altered conscious state. In a study which compared the self-reported QoL of patients with epilepsy to the QoL of patients with cardiogenic syncope [52], both epilepsy and syncope patients reported their QoL to be impaired to a similar degree, with no significant differences on any of the individual QoL parameters. As many forms of epilepsy and cardiogenic syncope share a final common path of consciousness impairment, these findings suggest that the unpredictable alteration of conscious states may be one of the main contributors to poor QoL, at least in a large subset of patients with epilepsy.

An understanding of how factors associated with consciousness impairment affect QoL could help the treatment and management of these patients. Although ideally the goal of treatment is seizure freedom in all patients, in many cases this is not achieved. We might hypothesise that significant improvements in QoL would be seen if seizures that induce consciousness impairments could be stopped.

### *1.4. Aims of the review*

At the outset, we attempted to review the existing evidence for the impact of alterations of consciousness (level and/or content) on QoL in patients with epilepsy. Interestingly, we found no study that has specifically focussed on the effect on QoL of the spectrum of seizure-induced impairments of consciousness in epilepsy. This reflects a general paucity of research into the phenomenology of epilepsy-induced alterations of consciousness, partially because of the lack of suitable research tools.

The deficiency of systematic studies concerning consciousness alterations and QoL in epilepsy limits our ability to shed light on this particular interaction. Until specifically-designed studies are available, we can only rely on secondary data obtained from current trials, and speculate about the relationship between ictal consciousness alterations and poor QoL in epilepsy. Bearing these limitations in mind, we focussed on studies of newer AEDs (Gabapentin (GBP), Lamotrigine (LTG), Levetiracetam (LEV), Oxcarbazepine (OXC), Tiagabine (TGB), Topiramate (TPM), Vigabatrin (VGP) and Zonisamide (ZNS)) and we set out to systematically review the evidence that a significant reduction (typically more than 50%) in seizures that induce alterations of consciousness, as a result of switching to one of the newer AEDs, leads to improvements in QoL. This may ultimately answer the questions whether a reduction of seizures that are typically accompanied by alterations of consciousness, leads to measurable improvements in QoL scores and if the improvements in QoL are solely attributed to reduced episodes of epilepsy-induced alterations of consciousness. Despite the limitations, we can begin to piece together a picture of the relationship between ictal alterations in consciousness and QoL based on these trials and it is to this that we now turn.

## 2. Search strategy and selection criteria

We identified references for this review by searching PubMed for the period 2000-2010 using the search terms 'epilepsy' or 'seizures' and 'quality of life', and 'Gabapentin', 'Lamotrigine', 'Levetiracetam', 'Oxcarbazepine', 'Tiagabine', 'Topiramate', 'Vigabatrin', 'Zonisamide' and 'quality of life in epilepsy'. All abstracts were read, and the articles found were further screened. Only articles in the English language were selected, and only those articles reporting data on adults (19+, so studies on children *only* were excluded). Studies on patients with intellectual disabilities or with concomitant progressive neurological disease or other condition that might have an impact on QoL were excluded. Finally, we were interested in studies that measured QoL by using a validated scale, i.e. psychometric testing had been carried out to look at validity, reliability etc. We also searched the Cochrane Library for systematic reviews. The reference lists of the selected articles with the above strategy were checked for additional materials when appropriate.

## 3. Results

We screened 247 article abstracts in total; the search yielded a number of review articles, two systematic reviews, three Cochrane reviews and 24 studies of newer AEDs reporting measuring quality of life. Of these, 11 studies were excluded from the systematic review mainly because they did not discriminate adequately between partial seizures (simple or complex) and thus

Table 1
Main studies reviewed

| No. | Author (publication year) | Seizure types | Newer AEDs | Sample size | QoL scales used |
|---|---|---|---|---|---|
| 1. | Schreiner et al. (2009) | Generalized, partial | TPM | 147 | QOLIE-10 |
| 2. | Wu et al. (2009) | Generalized, partial | LEV | 69 | QOLIE-10 |
| 3. | Kowalik et al. (2008) | Generalized, partial | TPM | 140 | QOLIE-10 |
| 4. | Marson et al. (SANAD, 2007) | Generalized | LTG, TPM | 716 | NEWQOL, IoE |
| 5. | Marson et al. (SANAD, 2007) | Partial | GBP, LTG, OXC, TPM | 1721 | NEWQOL, IoE |
| 6. | Berkovic et al. (2007) | Generalized | LEV | 164 | QOLIE-31-P |
| 7. | Steinhoff et al. (SKATE$^{TM}$, 2007) | Partial | LEV | 1541 | QOLIE-10-P |
| 8. | Somerville et al. (2007) | Partial | LEV | 152 | QOLIE-10-P |
| 9. | Cramer et al. (2004) | Partial | LTG | 143 | QOLIE-31 |
| 10. | Kaminow et al. (2003) | Generalized, partial | LTG | 122 | QOLIE-31 |
| 11. | Birberck et al. (2002) | Complex partial | VGP | 142 | QOLIE-31, QOLIE-89, SF-36 |
| 12. | Baker at al. (2002) | Generalized, partial | TPM | 209 | LSSS, SF-36 |
| 13. | Cramer at al. (2001) | Partial | TGB | 349 | QOLIE-89 |

GBP: Gabapentin, LTG: Lamotrigine, LEV: Levetiracetam, OXC: Oxcarbazepine, TGB: Tiagabine, TPM: Topiramate, VGP: Vigabatrin, ZNS: Zonisamide, QOLIE: Quality of Life in Epilepsy, NEWQOL: Newly Diagnosed Epilepsy Quality of Life, IoN: Impact of Epilepsy, QOLIE-10/31-P: Quality Of Life In Epilepsy-Patient weighted, SF-36: Short Form-36, LSSS: Liverpool Seizure Severity Scale.

useful conclusions for consciousness and quality of life in epilepsy could not be drawn. This left us with 13 main studies, which are described in Table 1 and discussed in the rest of this review.

Schreiner et al. reported an open-label, single arm, non-interventional study to explore the efficacy and tolerability of TPM, in patients moving from baseline monotherapy or combination therapy with valproic acid, because of inadequate seizure control and/or tolerability [54]. TPM was added initially to the existing AED regimen and based on the clinical judgment of the treating physician, a decision was made whether and when the existing antiepileptic scheme (particularly with valproate) could be withdrawn. In this multicenter study across Germany, 147 patients switched onto TPM, with 70% of them achieving monotherapy at study endpoint (20.3 weeks). Importantly, the majority of patients (more than 90%) had seizures types other than simple partial (complex partial, primary or secondary generalized tonic-clonic and/or absences), which is relevant to our enquiry about ictal consciousness alterations. At study endpoint, in 75% of patients, more than 50% reduction in seizure frequency was achieved and around 51% of the patients were seizure free. In parallel, the mean change from baseline QOLIE-10 score, showed a statistically significant improvement in QoL ($p < 0.001$) in all subscales. Another multicenter, open-label, non-interventional trial with a similar structure examined changing to TPM monotherapy from baseline monotherapy or combination therapy with carbamazepine (CBZ) or oxcarbazepine, for the same reasons as the previous study [40]. A total of 140 patients (with only a minority experiencing simple partial seizures) decided to switch to TPM, with 73% of them controlled on monotherapy. The average follow-up was 24 weeks, at which point 91% of the patients achieved more than 50% seizure reduction and 62% seizure freedom. By the end of the trial there was significant improvement in QoL compared to baseline ($p < 0.001$). These two studies, although single-armed, not randomized, and without a control group, provide a naturalistic setting concerning everyday clinical practice in seizure treatment. They also provide some evidence that significant reduction of seizures that induce alterations of consciousness, especially in patients with refractory epilepsy, lead to significant improvements in QoL. However, these results have to be interpreted with caution, since a proportion of the QoL improvement is attributed to better tolerability (fewer side effects) of the new antiepileptic drug.

The effects of TPM in an adjuvant setting on seizure severity and HRQoL in patients not well controlled on existing therapy, were assessed in an open-label, Canadian study [6]. The 209 patients enrolled in this 6-month study were evaluated at baseline and at the end of the treatment using two questionnaires: the Liverpool Seizure Severity Scale (LSSS) and the Short-Form 36 (SF-36). The former is a disease-specific measure of seizure severity, with many components directly related with many questions addressing aspects of ictal/post-ictal alterations of consciousness [7], while the latter is a generic measure of health status [64]. The majority of the patients had poorly-controlled seizures that impaired consciousness (only 60 patients had simple partial seizures). Overall, 44.3% of patients showed more than 50% reduction in seizures (from baseline to the last 8 weeks), and on the whole, the median reduction in seizure frequency was 40.9% ($p < 0.0001$). Apart from the reduced seizure frequency, patients demonstrated a notable mean reduction in LSSS (5.3, $p < 0.0001$), but there was no significant change in SF-36 scores. Again, this study was not a randomized, controlled clinical trial, and used a generic rather than an epilepsy-specific QoL scale, which failed to show a significant change. However, a *post-hoc* analysis showed that only patients experiencing more than 75% seizure reduction achieved a significant LSSS score reduction.

In a prospective, multicenter, open label study looking on the efficacy and the effects of LEV on cognitive and neuropsychological function and QoL [66], patients ($n = 94$, with 21 having simple partial seizures) who were prescribed LEV, either as an add-on or monotherapy, experienced significant seizure reduction at 1 year (39 patients were seizure free, while 68% had more than 50% reduction). However, there were only modest improvements in mean QOLIE-10 sub-scale scores. Notably, only patients with more than 50% seizure reduction had significant improvements in QOLIE-10 score on overall and total Quality of Life. The main conclusion is that a reduction in seizure frequency of 50% or less and the concomitant impairment of consciousness is not sufficient for a measurable improvement in QoL. An earlier, similar open-label trial, the SKATE study, which included only adults ($n = 1541$) with uncontrolled partial seizures treated with LEV as add-on therapy, resulted in 50.2% median reduction in seizure frequency from baseline (with 15.8% of patients achieving seizure freedom and 50.1% with more than 50% reduction) [59]. The QOLIE-10-P questionnaire was completed by patients in 10 of the 16 countries participating in this study (both at baseline and at the end-week 16). The results showed a significant improvement in QoL ($p < 0.001$), but varied widely from country to country, possibly pointing to cultural differences as another confounding factor in epilepsy, QoL and consciousness.

In a multicenter, double-blind trial, 164 patients with uncontrolled generalized tonic-clonic seizures associated with idiopathic generalized epilepsies, were randomized to adjunctive LEV therapy ($n = 80$) or placebo ($n = 84$) [9]. After a 4-week titration and 20-week evaluation period, 72.2% of patients on LEV had more than 50% reduction of seizure frequency per week, compared to 45.2% for placebo ($p < 0.001$; OR 3.28; 95% CI 1.68 to 6.38). The percentage achieving seizure freedom during the evaluation period was again greater for LEV compared to placebo (34.2% vs. 10.7%; $p < 0.001$). This reduction of seizure frequency led to improvements in QoL as assessed by QOLIE-31-P scale. Treatment with LEV not only resulted in greater improvements in terms of total score and most subscales compared to placebo, but also more patients (38.3%) actually reported important improvement since the start of the trial compared to the placebo group (28.6%). This study is of interest, in that the patient population included suffered from generalized tonic-clonic seizures, which cause the most profound alterations in consciousness.

Somerville et al. [57] reported an open-label clinical trial of LEV as adjuvant therapy in Australian patients ($n = 152$) with uncontrolled partial seizures. LEV reduced seizure frequency by at least half in 56.6% of patients and 12.5% became seizure free. In 25% of the patients that completed the QOLIE-10-P questionnaire ($n = 140$), there was a significant improvement in QoL. Not unexpectedly, seizure free patients had a far bigger increase in their total score, compared with those patients that experienced at least one seizure during the study course (19.3 vs. 4.0 increase in the score respectively, $p = 0.004$). A similar improvement in QoL was observed in patients who experienced at least one adverse event possibly related to the study drug ($n = 99$). These results indicate that the relationship between reduction in seizure frequency, decline in the number of episodes of consciousness impairment, and QoL is complex and is further complicated by the amount of reduction and the burdensomeness of the adverse effects of the AED regimens.

Cramer et al. [16] reported a study to assess the effect on patient-reported QoL after reduction from two drugs, to LTG monotherapy. After the conversion to monotherapy, changes from baseline to 12-week follow-up on the QOLIE-31 were compared with physicians' global change ratings and patient-reported health status. Of the 547 patients initially enrolled in the trial, analyses were based on the completers ($n = 143$) for whom outcome data were available. The majority of the patients had seizure types that are associated with ictal consciousness alterations (complex partial with or without secondary generalization or generalized tonic-clonic). An interesting finding emerges when we focus on the mean QOLIE-31 score change and subscale changes by seizure response. Although it seemed that patients with more than 50% reduction in seizure frequency or seizure freedom had improvement in the total score, further analysis showed that, in addition, patients with less than 50% reduction reported large improvements in some subscales (energy, cognition, medication effects), but not in others (seizure worry, overall QoL, emotional well-being, and social function). It is possible that the conversion to LTG monotherapy reduced the burden of adverse effects of polytherapy in this patient group, improving items such as energy, cognition, and medication effects. In parallel, as the seizure frequency and the effect of epilepsy-induced consciousness alteration on QoL was not significantly reduced, the score on relevant items such as seizure worry, overall QoL, emotional well-being, and social function was low. In other words, these subscales may have different sensitivities for different changes.

An open-label, United States multicenter study, evaluated the efficacy and tolerability of LTG monotherapy compared with monotherapy with conventional AEDs in patients converting from previous monotherapy due to inadequate seizure control or unacceptable side effects [34]. Patients ($n = 122$) were randomized 1:1 and received for 24 weeks monotherapy with LTG or monotherapy with either CBZ, phenytoin (PHT) or valproate. The percentages of patients with no seizures and with more than 50% seizure reduction were similar in the LTG and conventional therapy groups. However, the mean change from baseline QoL scores revealed statistical significant improvement ($p \leqslant 0.02$) for LTG compared with conventional therapy. This result suggests better tolerability of LTG, but precludes any useful conclusion for consciousness alteration and QoL.

Using data from a national (US) multicenter randomized control trial of VGB add-on therapy, Birbeck et al. [10] compared the change in HRQOL across groups having different levels of change in seizure frequency: 100%, 75–99%, 50–74% reduction, and 0–50% increase or decrease. The study population consisted of 134 adults with refractory complex partial seizures and the QoL was evaluated by using the QOLIE-31, QOLIE-89, and SF-36 instruments. Significant improvement in QoL was consistently shown only among those patients achieving seizure freedom (and thus experiencing no epilepsy-induced consciousness alterations), while QoL changes across the other categories were less positive. However, it is possible that the small numbers, especially for the 75–99% group ($n = 18$), limited the sensitivity of detecting a difference. These results raise several important issues. The patients were experiencing complex partial seizures, making this study relevant for consciousness alterations and underlie how detrimental this impairment can be, even with significant seizure reduction. These data suggest that for a significant improvement in QoL to occur, seizure freedom is necessary. Second, the study highlights important differences between the different QoL measures. For example, the SF-36 physical health summary score detected no improvement in the seizure-free group by ($p = 0.11$), while for the 75–99% reduction category, the overall QOLIE-89 epilepsy-targeted composite, showed some evidence of improvement ($p = 0.022$).

In 2007, Marson and colleagues reported an important trial comparing 'older versus new AEDs' [44,45]. This study, called the SANAD study, was an unblinded randomized controlled trial in hospital-based outpatient clinics in the UK and had two arms. Arm A comprised patients experiencing predominantly partial seizures ($n = 1721$) for whom CBZ was considered to be standard treatment, and they were randomized to receive CBZ, GBP, LTG, OXC, or TPM [44]. In arm B of the study, patients with generalized onset seizures ($n = 716$), for whom valproate was considered to be standard treatment, were randomly assigned to valproate, LTG, or TPM [45]. The primary outcomes were time to treatment failure, and time to 12-months remission. In addition, QoL outcomes were assessed using the NEWQOL (Newly Diagnosed Epilepsy Quality of Life) battery, a revised 12-item version of the Impact of Epilepsy scale (IoE) and EQ–5D (at entry to SANAD and annually thereafter, up to a maximum of 4 years post-randomization). In both arms, no or only few significant differences in QoL between treatment groups were recognized at 2 years. However, as the authors stated 'some trends in the data with regard to direction of treatment effects were evident'. Focussing on the pooled patient population data, significant differences both for achieving a positive (i.e. seizure remission) and a negative (i.e. treatment failure) clinical outcome emerge. In both arms, achieving a 12-month remission by 2-year follow-up was associated with a reduced likelihood of scoring negatively for global QoL (measured by the odds ratio), and also a small but significant improvement in QoL as measured by the EQ-5D. On the other hand, treatment failure was associated with poorer QoL (EQ-5D score) and increased likelihood of negative scoring for global QoL. An emerging theme in the context of these QoL assessments, as the authors reported, is that of response bias. Patients with poorer QoL at baseline assessment were less likely to return a QoL questionnaire at 2 years. Moreover, these non-responders were less likely to have achieved a 12-month seizure remission and more likely to have had a treatment failure.

Cramer et al. reported two simultaneously yet independent, randomized, double-blind trials, in which the efficacy, safety and impact on QoL of TGB addition to a standard AED (CBZ or PHT) were evaluated in patients with complex partial seizures (CBZ+PHT versus CBZ+TGB, and PHT+CBZ versus PHT+TGB) [15]. The novelty of this study, is that it tried to explore the impact on QoL during a short-term follow-up (16 weeks) and furthermore, to compare the changes in QoL (comparing subscales and total scores of QOLIE-89) among patients who responded to combination therapy ($\geqslant$ 50% seizure reduction), both within and between pairs of groups of each trial. In summary, although the PHT/CBZ group showed the highest response rate, unexpectedly, the QoL was not significantly better compared to the other groups. The TGB groups had the lowest response rates, but interestingly the PHT/TGB group showed the most 'promising' QoL results. In this particular group, a clustering of changes in three related cognitive subscales (attention-concentration, memory, and language) was noticed. Taken together, these results may give a hint that sometimes seizure improvement with parallel improvements in consciousness alterations (induced for example by complex partial seizures) may not wholly be responsible for QoL changes. As the sample of this study was small (especially the responders group), its findings are just exploratory and highlight the complicated and multifactorial nature of QoL changes.

## 4. Discussion

In this literature review, 13 studies of newer AEDs were found, in which the great majority of patients suffered from seizure types that are typically associated with alterations of consciousness. In all of the studies there was a significant seizure reduction ($\geqslant$ 50%) (and thus a parallel decline of the episodes of altered consciousness). The QoL of patients was measured using validated QoL tools. Our aim was to look for the association between seizure reduction as a reflection of reduced episodes of altered conscious state, and QoL. Such information is important as it can help delineate the ictal alterations of consciousness as a key driver of poor QoL in epilepsy. Many of the reviewed studies have provided evidence that a significant reduction or elimination of seizures (accompanied by the relevant improvements in ictal consciousness alterations), is associated with QoL improvements. The obvious question that emerges, is to what extent the reduction of episodes of consciousness impairment secondary to reduction in seizure frequency is responsible for improvements in QoL. Clearly, the relationship between seizure frequency, consciousness alterations and QoL is not a simple one and for many different reasons the conclusions of these studies must be viewed with much caution.

It is striking that in the 13 studies reported and reviewed, 11 different QoL instruments, both disease-specific and generic, are used. The diversity of QoL tools makes intra-study comparisons difficult, if not impossible. Currently, there is no consensus as to which instruments should be used to assess QoL in trials and in three Cochrane reviews identified [14,49,51] no attempt could be made to combine QoL results in a meta-analysis. This issue is even more complex when a statistically significant change is seen only in some scales, but not others (as was the case in two of the reported studies [15,16]). There are also differences in the psychometric properties of the scales e.g. the validity and sensitivity to change which makes comparisons difficult [19,37].

It is also important to consider the issue of data quality and statistical power. Five out of the thirteen reported studies were randomized controlled trials (RCTs), in which the highly controlled and monitored setting ensures that their results have a high degree of internal validity. On the other hand, this setting is more or less artificial, with restrictive inclusion and exclusion criteria, short-term follow-up and a high number of scheduled visits in order to collect information. If we add to these factors the possible trial effects, such as the positive impact of being closely monitored and given a new treatment, then the generalisability and the relevance of the QoL data to routine clinical practice may be less clear [55]. Many of these problems can be addressed by collecting QoL data in prospective studies that aim to examine the clinical effects and impact on QoL of AED regimens. Studies conducted in a so-called 'real world' setting, are becoming increasingly important for understanding the impact of therapeutic interventions. Eight out of the thirteen reviewed studies are not RCTs, but instead open-label prospective trials. The nature of these studies means that they do not have the same internal validity as RCTs, although they do have a high degree of external validity [55]. Importantly, the studies reviewed here were all multi-centre, meaning that their results may be generalisable. On the other hand, some of these trials were not sufficiently powered to show a difference in QoL scores and although conducted in a 'real world' background, the absence in some of a standardized patient population may limit the generalizability of the conclusions drawn. Furthermore, the researchers used different methods to analyse and report QoL data, and so caution is required when interpreting the results. But even if these methodological issues are satisfactorily addressed, the question remains: to what extent is an observed improvement in QoL scores attributable to an improved conscious state after a significant reduction in seizure frequency?

Another question, critical for clinical practice, is this: for a given contribution of ictal consciousness alterations in QoL, what percentage of reduction in seizure frequency is needed to show an improvement in QoL instruments? As discussed in the studies in the previous section, it seems that the most frequently used outcome used in clinical trials, i.e. 50% reduction in seizure frequency, may not be enough. Indeed, some data suggest that achievement of seizure freedom may be the key to improving QoL. Does this imply the ictal consciousness alterations pose a devastating threat, even when significantly reduced (including threat to employment, social and family function, sense of self, sense of control)? It would be surprising if the threat posed by consciousness impairment was not reflected somehow in the QoL profiles of people with epilepsy who experience them. Having said that, is interesting to look at the long-term QoL profile of epileptic patients that acquire seizure freedom and are consequently not affected by consciousness alterations. Most studies of QoL in seizure-free patients, suggest normal or near-normal functioning in many aspects of life [25,27,28, 58]. Some more recent studies though, have found an impaired QoL (decline in overall QoL, in social and physical function, in symptoms of depression, health concerns and low self-esteem) even in seizure-free patients, and notably, to a higher degree that would 'normally' be expected [2,3,25,26,39]. These unexpected findings show that QoL in patients with epilepsy is determined by more than seizure control and alterations of consciousness [53]. After all, epilepsy is a multifaceted chronic disorder with diverse, complex and versatile effects on the well-being of the patient. Many factors affect QoL and this hinders the drawing of firm conclusions concerning the specific contribution of consciousness alterations (especially when the available studies are not actually measuring consciousness impairment).

The available evidence suggests that, of the many variables associated with QoL, seizure activity (with consciousness alterations) and adverse effects of AED treatment [3] show the strongest associations, with other epilepsy and non-epilepsy factors being weaker [29]. A growing literature is addressing the contribution of psychiatric status (particularly mood, anxiety, depression) to the perceived QoL in patients with epilepsy [18, 29,31,33,35,38,47,53,56,68]. Epilepsy carries a risk of unpredictable alteration and loss of consciousness and a complex pattern of wider consequences. To complicate things even more, some AEDs may have beneficial effects on mood, while others have adverse effects.

## 5. Conclusions

Despite the differences between seizure types, many of them impair consciousness and alter the patient's private experiences and well-being. In this article, we have discussed the possible interactions of alterations and loss of consciousness with QoL in patients with epilepsy and we draw on available literature of newer AEDs studies, to propose likely associations and confounding factors. Although these studies suggest the direction of the association, the methodological difficulties mean that we do not have a clear picture of the impact of alterations of consciousness on QoL in epilepsy. The methodological issues should be addressed in future research.

The last decade has sometimes been called 'a renaissance of consciousness studies'. Well-designed research using validated tools to measure alterations of consciousness in a defined selected population of patients with epilepsy is needed, to fully understand the impact of alterations of consciousness on QoL in epilepsy. Research tools have now been developed which will facilitate research, such as the Ictal Consciousness Inventory (ICI), which allows comprehensive assessment and quantification of the full spectrum of ictal alterations of consciousness [12,13,48]. The use of this 20-item self-report battery, as an adjunct to QoL measures in the context of a drug trial, can help us understand how alterations of the ictal conscious state can influence the QoL of patients.
